# OA26 Atypical Multisystem Inflammation related to infection or just plain old polyarteritis nodosa?

**DOI:** 10.1093/rap/rkae117.026

**Published:** 2024-11-05

**Authors:** Enas Alyaldin, Peter Bale

**Affiliations:** Mid and South Essex NHS Foundation Trust, Basildon, United Kingdom; Cambridge University Hospital NHS Foundation Trust, Cambridge, United Kingdom

## Abstract

**Introduction:**

We present the case of a 5-year-old boy with episodes of pyrexia of unknown origin associated with polyarthritis, central neuroinflammation and mucocutaneous manifestations. His skin biopsy demonstrated a nodular vasculitis which could be consistent with polyarteritis nodosa. Interestingly though, he had additional atypical rashes, ocular inflammation and evidence of acute and past streptococcal infection and his fever and CRP normalised prior to corticosteroid initiation.

**Case description:**

A five-year-old British African male presented with five-week history of persistent fever, headaches, joint pain and bilateral eye pain. He was transferred to a tertiary paediatric infectious disease’s unit due to unrelenting fever with no fixed pattern, despite nine days of broad-spectrum intravenous antibiotics. He was diagnosed with neuroinflammation of uncertain aetiology, evidenced by leptomeningeal enhancement and bilateral papillitis on brain MRI. CSF microbiology and autoimmune profiles were negative. There was no evidence of primary malignancy. The fever resolved with pulsed IV methylprednisolone and the child was discharged home on a weaning regime of oral prednisolone. After stopping steroid treatment, he represented with high-grade fever, limping, anterior uveitis and new-onset non tender cutaneous lesions, plaques over eyelids and cheek and annular lesions over arms, legs and buttocks. Repeat MRI of the brain and spine showed resolution of previous neuroinflammation.

Blood tests revealed raised ANA, IgG and ASOT titre, and his throat swab was positive for Group A Streptococcus. He completed 10 days of intravenous antibiotics and oral high dose ibuprofen (10 mg/kg TDS). On review by tertiary paediatric rheumatology, he was identified to have pleomorphic skin rash and subcutaneous nodules. Skin and deep tissue biopsy confirmed lobular and septal panniculitis with necrotising vasculitis, consistent with a nodular vasculitis. Treatment with high-dose steroid and mycophenolate mofetil (MMF) was initiated, but his CRP and fever had resolved prior to his first dose. He experienced improvement in systemic symptoms and arthritis; however, skin lesions persisted, with appearance of new lesions and hyperpigmentation, on reduction of steroid dose. His course was complicated by acute varicella infection, requiring oral aciclovir. Further treatment trials included topical tacrolimus and topical corticosteroids for his skin lesions. At one biopsy site, he experienced significant delay in wound healing. The genetic panel for primary immunodeficiency panel (R15) demonstrated no pathological variants.

**Discussion:**

This case report highlights that, whilst the biopsy may be consistent with nodular vasculitis, such as PAN, the clinical manifestations are not perfectly aligned. Discussion within the rheumatology community would support optimal patient care.

Childhood-onset Polyarteritis Nodosa (c-PAN) is a rare systemic vasculitis. Although the aetiology is unknown, associations with infections like group A streptococcus and hepatitis B suggest a postinfectious autoimmune response. There are no clear genetic links to PAN, but PAN-like vasculitis is part of reported monogenic autoinflammatory conditions.

Key atypical features in this case include uveitis at presentation, nonspecific central neuroinflammation, unusual cutaneous lesion morphology, improvement of acute phase reactants before immunomodulatory treatment, and delayed biopsy wound healing. While ocular findings in PAN may include episcleritis, scleritis, keratitis, retinal vascular occlusions, and ocular ischemic syndrome, uveitis has not been reported in c-PAN.

The patient also showed nonspecific inflammatory changes on initial MRI. Neurological involvement in c-PAN typically includes cerebrovascular accidents, cranial nerve palsies, and mononeuritis multiplex, with meningeal involvement being less common (reported 4% in a study of 69 children). Additionally, the patient experienced delayed wound healing following the biopsy, which is undocumented in c-PAN, but may be related to immune modulating treatment.

A positive streptococcal throat swab could suggest an immune reaction as part of bacterial tonsilitis, or Acute Rheumatic Fever. However, he did not meet Jones Criteria, there was no response to prolonged antibiotics and the chronic remitting nature made these less likely.

An autoinflammatory disorder, Deficiency of Adenosine Deaminase 2 (DADA2), was also considered but he had no evidence of livedo racemosa, strokes and no CECR1 mutation was identified.

Our case has been discussed within a multidisciplinary team and we speculate that it could represent a previously undefined immune sensitivity within the autoimmune, autoinflammatory and immune deficiency spectrum giving an exaggerated response to infection.

**Key learning points:**

• This case presents a significant diagnostic challenge due to its atypical clinical features and inconclusive extensive investigations, including immunologic testing, tissue biopsy, and genetic analysis.

• While the patient’s symptoms improved with high-dose steroids and MMF, the recurrence of symptoms upon tapering steroids underscores the persistent inflammatory nature of the disease.

• The detection of group A Streptococcus in the patient’s throat swab suggests a potential postinfectious autoimmune trigger, highlighting the role infections can play in the pathogenesis of vasculitis. The negative results from the R15 Primary Immunodeficiency panel suggest that more advanced genetic and immunologic testing may be beneficial.

• We invite discussion on whether additional genetic or immunologic testing could optimise this patient’s care and contribute to the broader understanding of atypical vasculitis syndromes.

